# Supplementary material for: Confronting SARS-CoV-2 Infection: Patients’ Experience in the First Pandemic Wave—Cross-Sectional Study
Source: Int J Environ Res Public Health. 2022 Oct 5;19(19):12743. doi: 10.3390/ijerph191912743 (PMC9566657; doi:10.3390/ijerph191912743)
Supplement: Supplementary file 1 [file ijerph-19-12743-s001.zip › ijerph-1949173-supplementary.pdf]

Supplementary material

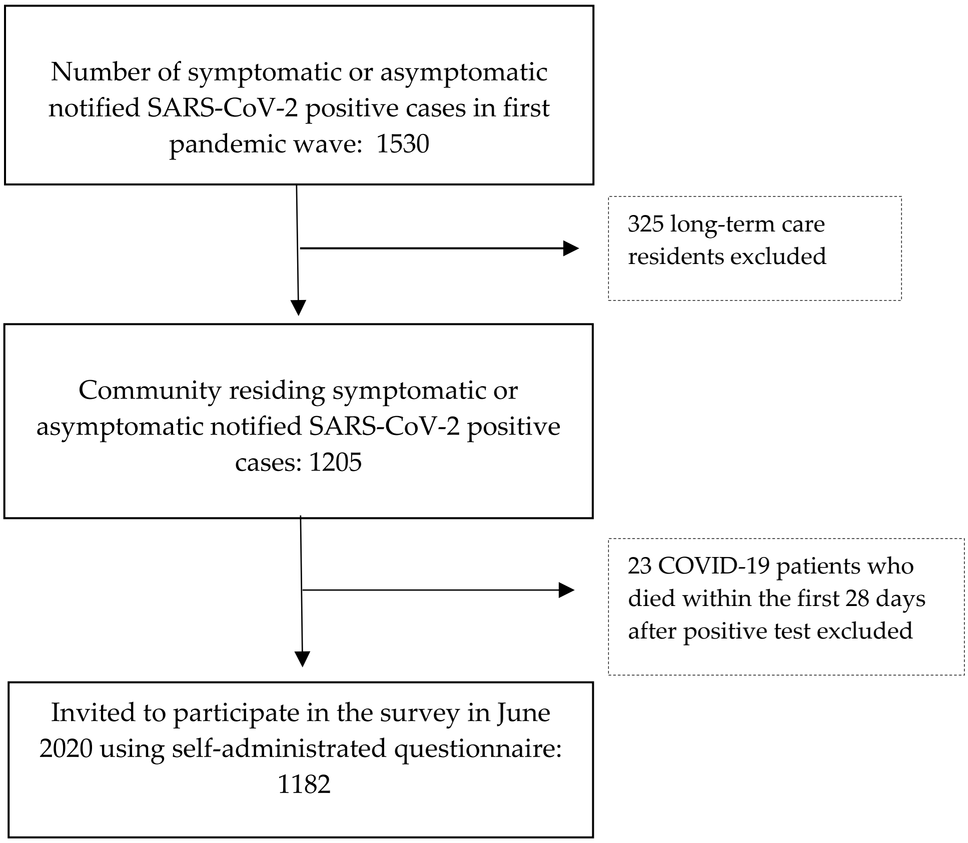

Figure S1. Participants selection for the self-administrated survey regarding experience with SARS-CoV-2 infection in the first pandemic wave (from 4.3.2020 to 31.5.2020) in Slovenia.

QUESTIONNAIRE ON FIRST PANDEMIC COVID-19 EXPERIENCE

You are kindly invited to answer the questions by crossing the box next to the answer that applies to you. If you do not want to answer a certain question, you can skip it. If the questionnaire was addressed to a child, parents should fill in.
Date of completing the questionnaire_______________________________

WHAT IS YOUR HIGHEST LEVEL OF EDUCATION ACHIEVED?
☐ Incomplete primary school education
☐ Primary school education
☐ Secondary vocational education
☐ Secondary education (gymnasium)
☐ Higher professional education (including the 1st Bologna level)
☐ Higher university education (includes the 2nd Bologna level)
☐ Specialization, master's degree, doctorate

YOUR GENERAL HEALTH CONDITION
Mark all your health issues.
☐ High blood pressure
☐ Diabetes
☐ Cardiovascular disease
☐ Asthma
☐ Chronic obstructive pulmonary disease (COPD)
☐ Other lung diseases
☐ Kidney disease
☐ Liver disease
☐ Cancer
☐ Diseases that weakened your immune system. Specify the disease.
☐ Other chronic diseases. Specify the disease.
☐ Pregnancy
☐ Smoker
☐ Frequent consumption of alcohol
What medicines do you take for the above listed diseases?
Have you used ibuprofen during your COVID-19 illness? ☐ Yes ☐ No
Have you used any other medicines to relieve the symptoms of COVID-19?
☐ Yes Specify which.
☐ No

THE COURSE OF YOUR DISEASE
1. After the confirmation of COVID-19, did you need help of a doctor due to the disease?
☐ No
☐ Yes
If yes, please explain why:
☐ I needed additional clarifications and instructions regarding COVID-19
☐ Due to the deterioration of my health, I needed a telephone consultation
☐ Due to the deterioration of my health, I needed a visit in an outpatient clinic
☐ I needed a referral to the hospital
☐ I have been in contact with my doctor regarding sick leave/end of sick leave
☐ Other:
☐ No

Have you been treated in the hospital for COVID-19?
☐ Yes Indicate the number of days:
Indicate the date of hospitalization: from_ to_
☐ No
Did you need oxygen while being treated for COVID-19 in the hospital?
☐ Yes ☐ No ☐ I don't know
Did you need respirator?
☐ Yes ☐ No ☐ I don't know

What symptoms did you have during COVID-19? (mark all symptoms you had)
☐ I have not experienced any symptoms
☐ Increased body temperature, indicate the highest measured temperature:
☐ Cough
☐ Sore throat
☐ Shortness of breath
☐ Difficulty with breathing
☐ Headache
☐ Diarrhea
☐ Pain in the muscles
☐ Loss of smell and/or taste
☐ Fatigue
☐ Other:

ASSESSMENT OF YOUR HEALTH CONDITION
When answering the following two questions, please consider your state of health on the 15th day after the onset of the illness.
Which symptoms of COVID-19 were present on the 15th day after the onset of illness? (mark all symptoms you had)
☐ I have not experienced any symptoms
☐ Increased body temperature
☐ Cough
☐ Sore throat
☐ Shortness of breath
☐ Difficulty with breathing
☐ Headache
☐ Diarrhea
☐ Pain in the muscles
☐ Loss of smell and/or taste
☐ Fatigue
☐ Other:

On the 15th day, were you able to perform your usual daily activities?
☐ Yes, completely as before the illness
☐ I wasn't completely healthy, but I was mostly able to do my daily activities and didn't need help
☐ I covered my basic needs and chores (e.g. I brushed my teeth, do other hygienic chores, I ate by myself etc.), other every day chores were done by others in my household or by my friends
☐ I spent most of the day in bed and needed help from family members or friends
☐ I was in the hospital

YOUR LIVING CONDITIONS
Where do you live?

☐ Single-family house
☐ Multi-apartment house
☐ Multi-room apartment in a block of flats
☐ Studio apartment, one-room apartment in a block of flats
☐ Other:

Do you have a pet?
☐ Yes   Specify which:
☐ No

Given your living conditions, were you able to secure separate accommodation from the others in the household?
☐ I live alone
☐ Yes, I had my own room
☐ Yes, I moved to another location
☐ No
☐ I was treated in the hospital
☐ Other:

YOUR FEELINGS DURING COVID-19
How did you feel when you found out that you were infected with SARS-CoV-2?
☐ Sadness
☐ Fear
☐ Worry
☐ Indifference
☐ Anger
☐ Blame
☐ Confusion
☐ Other:

How did you feel when the infection lasted for several days?
☐ Sadness
☐ Fear
☐ Worry
☐ Indifference
☐ Anger
☐ Blame
☐ Confusion
☐ Other:

Did you have satisfactory support from people who are close to you (family members, relatives, friends)?
☐ Yes
☐ No
☐ Partly

Did you have satisfactory support from the wider community (colleagues, acquaintances)?
☐ Yes
☐ No
☐ Partly

Did you have satisfactory support from the health service (your physician, other medical staff)?
☐ Yes
☐ No
☐ Partially

Please provide an explanation:

What problems did you encounter during the first 15 days of your illness? (you can mark several answers)
☐ I was worried about my health
☐ I was worried about the health of my loved ones
☐ With a lack of food, medicine and other necessities
☐ With lack of physical activity
☐ I felt lonely
☐ With problems in relationships between family members
☐ I was worried about the loss of income
☐ I was worried about the future of the country or society
☐ Other:

Have you spoken openly about your illness with friends and colleagues?
☐ Yes
☐ No
☐ Partly

8. Have you felt stigmatized/marked/judged due to the disease of COVID-19?
☐ Yes
☐ No
☐ Partly
Please provide an explanation:

ORGANIZING YOUR LIFE
Who helped you with food, medicine and other necessities during the isolation? (you can mark several answers)
☐ I didn't need help
☐ Close family members
☐ Family members with whom I do not live together
☐ Friends
☐ Neighbors
☐ Volunteers
☐ Red Cross
☐ Municipal civil protection
☐ Other:

Where did you look for information regarding COVID-19 during your illness? (you can mark several answers)
☐ Television
☐ Radio
☐ Telephone number 040 18 18 (Government Communications Office – COVID-19 free information)
☐ Social networks
☐ NIPH (National Institute of Public Health) website
☐ Other domestic web pages

☐ Foreign web pages
☐ Friends, acquaintances
☐ Medical staff (your physician, infectious disease specialist, epidemiologist)
☐ Scientific literature
☐ I didn't need any additional information
☐ Other:

Which source of information about COVID-19 was most important to you during the illness? (mark only 1 answer)
☐ Television
☐ Radio
☐ Telephone number 040 18 18 (Government Communications Office – COVID-19 free information)
☐ Social networks
☐ NIPH (National Institute of Public Health) website
☐ Other domestic web pages
☐ Foreign web pages
☐ Friends, acquaintances
☐ Medical staff (personal physician, infectious disease specialist, epidemiologist)
☐ Scientific literature
☐ I didn't need any additional information
☐ Other:

PROPOSALS
Thank you for completing the questionnaire. Please write down if you want to tell us anything or if you have any suggestions for improving the functioning of the health and non-health sectors regarding the COVID-19.
